# The protective effect of caffeic acid on global cerebral ischemia-reperfusion injury in rats

**DOI:** 10.1186/s12993-015-0064-x

**Published:** 2015-04-18

**Authors:** Guojuan Liang, Bin Shi, Weinan Luo, Junqing Yang

**Affiliations:** Department of Pharmacology, Chongqing Key Laboratory of Biochemistry and Molecular Pharmacology, Chongqing Medical University, Medical College Rd. No 1, Chongqing, 400016 P. R. China

**Keywords:** Global cerebral ischemia-reperfusion, Caffeic acid, 5-LO, Brain injury

## Abstract

Ischemic stroke is a major cause of death and disability all over the world. Ischemic stroke results from a temporary or permanent reduction of cerebral blood flow that leads to functional and structural damage in different brain regions. Despite decades of intense research, the beneficial treatment of stroke remains limited. In light of this, the search for effective means ameliorating cerebral ischemia-reperfusion injury (CIRI) is one of the major problems of experimental medicine and biology. Recently, the 5-Lipoxygenase (5-LO, a key enzyme metabolizing arachidonic acid to produce leukotrienes) inhibitors have been showed to protect brain against ischemic damage in animal model of cerebral ischemia. Caffeic acid, an inhibitor of 5-LO, is a phenolic compound widely distributed in medicinal plants. The aim of this study was to investigate the effect of caffeic acid on global cerebral ischemia-reperfusion injury in rats. The study was carried out on 45 rats that were randomly divided into five groups: the sham group (n = 9), I/R non-treated group (n = 9), I/R-caffeic acid group (10 mg · kg^−1^) (n = 9), I/R-caffeic acid group (30 mg · kg^−1^) (n = 9) and I/R-caffeic acid group (50 mg · kg^−1^) (n = 9). Global cerebral ischemia was induced by bilateral carotid artery occlusion for 20 min followed by reperfusion. Spatial learning and memory was evaluated using Morris water maze. Histopathological changes of hippocampus neurons was observed using HE staining. Superoxide dismutase (SOD, the antioxidant enzyme) activities and malondialdehyde (MDA, an oxidative stress biomarker) contents were detected. NF-κBp65 expression was detected by the methods of immunohistochemistry. Caffeic acid markedly reduced the escape latency, relieved hippocampal neurons injury and increased neuron count compared with those of I/R non-treated rat. NF-κBp65 expression and MDA content decreased significantly, and SOD activities increased significantly in hippocampus. Compared with sham group, 5-LO expression increase significantly in I/R non-treated group rat, and caffeic acid markedly reduced 5-LO expression. The results of the study suggest that caffeic acid has a significant protective effect on global cerebral ischemia-reperfusion injury in rats. The neuroprotective effects is likely to be mediated through the inhibition of 5-LO.

## Introduction

Ischemic stroke is a major cause of death and disability all over the world [1]. Indeed, it is the third leading cause of death, after heart disease and cancer, and the leading cause of long-term disability in major industrialized countries [2]. Ischemic stroke results from a temporary or permanent reduction of cerebral blood flow that leads to functional and structural damage in different brain regions. Cellular damage occurs during ischemia [3,4] and reperfusion [5,6]. Despite decades of intense research, the beneficial treatment of stroke remains limited [7]. In light of this, the search for effective means ameliorating cerebral ischemia-reperfusion injury (CIRI) is one of the major problems of experimental medicine and biology [8].

Currently, the mechanisms of neuronal injury and death induced by cerebral ischemia-reperfusion (I/R) are not completely known. Recently, 5-Lipoxygenase (5-LO), a key enzyme metabolizing arachidonic acid to produce leukotrienes [9,10], has been reported to be involved in brain injury [11,12]. 5-LO expression is increased and leukotriene contents are elevated in the ischemic brain [13-16], indicating a role of 5-LO in cerebral ischemia. The importance of 5-LO in stroke has been proven by the reports indicating that the gene encoding 5-LO activating protein confers risk of stroke [17-19]. The 5-LO inhibitors have been showed to protect brain against ischemic damage in animal model of cerebral ischemia [20-23].

Caffeic acid (3,4-dihydroxycinnamic acid, CA) is a phenolic compound widely distributed in medicinal plants, including fruits, vegetables, wine, coffee and olive oil, among others, and is therefore present in human plasma in a diet dependent concentration [24-26]. CA has been shown to be an inhibitor of 5-LO [27,28], and it is an intriguing compound because it possesses various pharmacological activities, including antioxidants [29-32], metal chelating agent [33], antihypertensive [34], antifibrosis [35,36], antiviral [37-39], anti-cancer [40], antidepressive [41,42], anti-inflammatory [43-45] and antidiabetic [46,47] properties. Recently neuroprotective effect of CA has also been suggested [48-51]. Previous studies have related an important protective effect of CA on focal cerebral ischemia-reperfusion injury [12]. However, its neuroprotective effect on global cerebral ischemia-reperfusion injury remains unclear. Thus, the aim of this study was to investigate the effect of CA on global cerebral ischemia-reperfusion injury in rat brain with biochemical and histological analysis.

## Materials and methods

### Source of chemicals

Caffeic acid was purchased from Sigma Chemical Company (St. Louis, MO, USA). All other chemicals and biochemicals used for the experiments were of analytical grade obtained from local firms.

### Animals and experimental procedures

A total of 45 Male Sprague–Dawley rats (200–250 g) were purchased from the Laboratory Animal Center of Chongqing Medical University, Chongqing, China. Rats were housed in standard conditions of 25 ± 1°C, 50 ± 2% humidity, with 12/12 h light/dark cycles (light from 8:00–20:00) and ad libitum access to water and standard pellet food. All experiments were carried out in accordance with the National Institutes of Health Guide for the Care and Use of Laboratory Animals, and were approved by the Medical Ethics Committee of Chongqing Medical University. The animal application approval number was SCXK2007-0001, which was given by the Chongqing Science and Technology Commission in September 2007.

### Experimental design

Rats were divided into five groups: the sham group (n = 9), I/R non-treated group (n = 9), I/R-caffeic acid group (10 mg · kg^−1^) (n = 9), I/R-caffeic acid group (30 mg · kg^−1^) (n = 9) and I/R-caffeic acid group (50 mg · kg^−1^) (n = 9). In I/R-caffeic acid groups, the rats were administrated caffeic acid at 10, 30, 50 mg · kg^−1^ (prepared with 0.3% sodium carboxymethyl cellulose) by intraperitoneal injection at 30 min prior to ischemia. The sham group and I/R group were treated with an equal volume of 0.3% sodium carboxymethyl cellulose.

### Induction of global cerebral I/R model

Rats were anesthetized by intraperitoneal injection of chloral hydrate (400 mg/kg), and fixed in a supine position. Global cerebral ischemia was induced as previously described [52,53]. A midline incision was made in the neck, after that the incision was extended 1 cm to the right. Then both common carotid arteries and the right common jugular vein were exposed carefully by blunt dissection. The distal end of the common jugular vein was ligated following 2 ml heparinized saline (100 mL 0.9% saline containing heparin (250 U)) were perfused. The blood accounting for about 30 percent of the total blood volumes were taken from the right common jugular vein leading to hypotension. Global cerebral ischemia was induced by bilateral clamping of the common carotid arteries combined with hypotension. After ischemia for 20 min, the artery clamps were removed, and the extracted blood was reinfused. Rats in the sham group were subjected to the same operation as above, excepted for the bilateral carotid artery occlusion and hemospasia from the right common jugular vein.

### Morris water maze test

At the 8th day after global cerebral I/R, the rats’ spatial learning and memory was assessed by the Morris water maze test, which consisted of 4-day training (at about the same time (5:00 – 10:00 pm)) and 1-day test. This was carried out as described previously [54,55] with some modifications. Rat spatial learning and memory were tested using a DMS-2 Morris water maze (Institute of Materia Medica, Chinese Academy of Medical Sciences, Beijing, China) (150 cm diameter, 50 cm height) filled to a depth of 40 cm with water maintained at 24-25°C. An invisible platform (25 cm in diameter) was submerged 2 cm below the water surface and placed at the midpoint of one quadrant. During the training, the rats (n = 9 in each group) were sent into the water separately and forced to seek the platform (safety island) by swimming. If the rats found the platform within 180 s, it was allowed to stay on the platform for 10 s, otherwise, it was gently navigated to the platform by hand for 10 s. The rats were subjected to four trials with a 1-h interval between trials every day. On the fifth day, the platform was removed and the rat was put into the water at the same place as before. The time for the rat to pass through the place, where the platform was previously placed, was called as the time for exploring platform. The time for exploring platform was recorded using a video tracking device integrated with Morris water maze test apparatus. The cut out time was 180 s.

### Histopathological examination

After spatial memory evaluation, rats (n = 3) in each group were anesthetized with 4% chloral hydrate (400 mg/kg, ip). Then, the rats were perfused transcardially with 4% paraformaldehyde after a pre-wash with 100 mL 0.9% saline containing heparin (250 U). The brain tissue was isolated and fixed in 4% paraformaldehyde for 3–5 days. Then the brain tissue was cutted into thin blocks, and fixing in 95% ethanol and carrying out the subsequent dehydration and clearing at refrigerator temperatures (4°C). Thereafter, embedding in paraffin, and then coronal sections of 5 *μ*m in thickness were cut from each block. The sections were stained with hematoxylin and eosin (H&E). The histomorphology of neurons in each rat hippocampus was observed by light microscopy. For assessment of cell counts from H&E-stained sections, 10 consecutive high-power fields were sampled from the dorsal hippocampal CA1 subfield. Pyramidal cells with a distinct nucleus and nucleolus were regarded as intact, while neurons showing typical signs of dark staining, shrinkage and dysmorphic shape were considered as damaged. Counts of intact neurons were performed from the ischemic and sham brains using a microscope at 400 x magnification, and the extent of cell death was estimated, expressed as percentage for each hippocampal region.

### Reverse transcription-polymerase chain reaction (RT-PCR)

After spatial memory evaluation, rats (n = 3) in each group were sacrificed (total of 15 rats). Brains were removed, and the hippocampi were separated. To determine 5-LO mRNA expression after ischemia, total RNA was extracted from the cerebral hippocampus of rats using Trizol reagents following the procedures described in manufacturer’s instructions. Total RNA integrity was identified using 0.8% agarose gel electrophoresis, and RNA purity and concentration were determined using ultraviolet spectrophotometry. Total RNA concentration was adjusted to 1.0 μg/μL.

For the cDNA synthesis, the total reaction volume for RT was 20 *μ*L, including 4 *μ*L 5 × PrimeScriptTM Buffer, 2 *μ*L 10 mM dNTP, 0.5 *μ*L 40 U/*μ*L RNase inhibitor, 2 *μ*L 2.5 *μ*M oligo (dT) Primer, 8 *μ*g of RNA template, 0.5 *μ*L PrimeScriptTM RTase and 5 *μ*L RNase-free dH2O. The mixture was incubated at 42°C for 20 min, 95°C for 5 min, 4°C for 15 min to inactivate the reverse transcriptase. PCR was performed as follows: 2.5 μL cDNA mixture was reacted in the reaction buffer (25 μL) containing 12.5 μL Premix Taq, 0.5 *μ*L forward primers, 0.5 *μ*L reverse primers and 9 *μ*L sterile double-distilled water. The thermal cycling conditions for the PCR involved an initial denaturation step at 94°C for 3 min followed by 35 cycles at 94°C for 30 s, 55°C for 30 s and 72°C for 45 s, finally stopped at 72°C for 5 min. PCR products were separated by 2% agarose gel electrophoresis and visualized by ethidium bromide staining. The absorbance value of 5-LO and β-actin mRNA was measured using a Bio-Rad gel imag-ing analysis system (Bio-Rad Laboratories). The absorbance ratio of 5-LO mRNA to β-actin mRNA was defined as the 5-LO mRNA relative quantity. The primer sequences for 5-LO were: forward 5′-TGTACCCAGAGGAGCAT-3′and reverse 5′-ACGGCAAAGCCTTAGAT-3′; which results in a PCR product of 177 bp. Those for β-actin were: forward 5′-AAGATCCTGACCGAGCGTGG-3′ and reverse 5′-CAGCACTGTGTTGGCATAGAGG-3′; which results in a PCR product of 318 bp.

### Western blotting

After spatial memory evaluation, rats (n = 3) in each group were sacrificed, and brains were removed. Rat hippocampus were chopped into small pieces, homogenized in 0.5 ml of RIPA buffer. The dissolved proteins were collected from the supernatant after centrifugation at 12 000 × g at 4°C for 5 min. The supernatant was collected for Western blotting analysis. The protein concentration of the supernatant were determined with the BCA Protein Assay reagent kit following the manufacturer’s protocols. Equal amounts of protein samples were separated by SDS/PAGE and then transferred to PVDF membranes. The membranes were blocked with 5% skim milk for 2 h and incubated with rabbit anti-5-LO (1: 300; Beijing Boaosen Biotechnology Co., Ltd., Beijing, China) and rabbit anti-β-actin polyclonal antibody (1: 1 000; Beijing Boaosen Biotech-nology Co., Ltd., Beijing, China) at 4°C overnight. After washing with TBST, the membranes were incubated with a horseradish peroxidase-conjugated secondary antibody (1:2000) for 2 h at 37°C. Finally, the membranes were reacted with the ECL reagents and exposed on an X-ray film. The optical densities of 5-LO and β-actin bands on the X-ray film was measured using a Bio-Rad gel imaging analysis system (Bio-Rad Laboratories). The results were expressed as 5-LO/β-actin ratios.

### Biochemistry

After spatial memory evaluation, 3 rats from each group were sacrificed and the hippocampi were dissected. The hippocampus (100 mg) was homogenized with saline (tissue: saline = 1:9). The malondialdehyde (MDA) content and the activities of superoxide dismutase (SOD) were measured using the biochemistry assay kit (Jiancheng Bioengineering Ltd, Nanjing, China) according to the manufacturer’s manual. The protein content of samples was measured using biuret reaction.

### NF-κBp65 immunohistochemistry

The paraffin sections (the same as those for H&E staining) were used for immunohistochemical assays following the manufacturer’s protocol: The sections were deparaffinized in xylene and hydrated through a series of graded ethanol, and washed thrice with PBS (0.02 mmol/L, pH 7.4) for 3 min. The sections were incubated with 3% H_2_O_2_ for 10 min. After the section was rinsed for 3 min × 3 with PBS, the rehabilitation of antigen was performed by microwave oven. Then the sections were incubated with a polyclonal antibody to NF-κBp65, (diluted 1:100) overnight at 4°C. The sections were rinsed for 3 min × 3 with PBS before incubation with biotinylated goat anti-rabbit IgG antibody for NF-κBp65, for 40 min at 37°C, and incubated with streptavidin for 20 min,and then rinsed for another 3 min × 3 with PBS before reaction with DAB solution. The sections were counterstained with hematoxylin and then observed under a microscope. The absorbance value of 5-LO and β-actin mRNA was measured using a Bio-Rad gel imaging analysis system (Bio-Rad Laboratories). The absorbance ratio of 5-LO mRNA to β-actin mRNA was defined as the 5-LO mRNA relative quantity.

### Statistical analysis

Experimental data are expressed as Means ± SD. Analysis of variance was performed using the SPSS 17.0 statistical software package (SPSS, Chicago, IL, USA), with significance of differences within-group and between-group analyzed by Dunnett’s test. Statistical significance was considered when P < 0.05.

## Results

### Changes in learning and memory function in rats

There was a significant difference between all groups for the time spent in finding platform. Compared with the sham group, the time taken to find platform was significantly longer in the I/R non-treated group (*P* < 0.01). Compared with the I/R non-treated group, the latency to find platform was significantly shortened in low- and high-dose caffeic acid groups, the shortened platform latency was most evident in the I/R-caffeic acid group (50 mg · kg^−1^) (*P* < 0.01) (Table 1).

**Table 1:** **Exploring time of rats (** $\overline{x}\pm s$ **,** ***n*** **= 9)**

	**Exploring time(s)**				
	**d1**	**d2**	**d3**	**d4**	**d5**
Sham	112.46 ± 16.14	72.33 ± 15.20^aa^	42.48 ± 3.50^b^	23.25 ± 2.30^c^	18.78 ± 2.70^d^
I/R	166.79 ± 23.7^**^	129.05 ± 8.3^**a^	77.53 ± 2.3^**bb^	65.73 ± 8.3^**c^	42.48 ± 3.50^**d^
I/R-caffeic acid (10 mg · kg^−1^)	164.22 ± 19.6	94.16 ± 25.45^#aa^	53.95 ± 5.3^#bb^	45.35 ± 5.3^#c^	26.04 ± 3.19^##d^
I/R-caffeic acid (30 mg · kg^−1^)	148.84 ± 17.6^#^	89.11 ± 9.88^#aa^	46.71 ± 6.5^#bb^	31.88 ± 3.9^##cc^	23.70 ± 1.49^##^
I/R-caffeic acid (50 mg · kg^−1^)	126.48 ± 3.26^#^	70.99 ± 9.63^##aa^	43.95 ± 4.1^##b^	25.01 ± 0.4^##cc^	20.23 ± 8.85^##^

^**^ *P* < 0.01 *vs* Sham group; ^#^ *P* <0.05, ^##^ *P* < 0.01 *vs* I/R group; ^a^P < 0.05, ^aa^P < 0.01 *vs* d1; ^b^P < 0.05, ^bb^P < 0.01 *vs* d2; ^c^P < 0.05, ^cc^P < 0.01 *vs* d3; ^d^P < 0.05 *vs* d4; d1-d4: 4-day training; d5: 1-day test.

### Morphological changes of rat hippocampus

**Figure 1:**
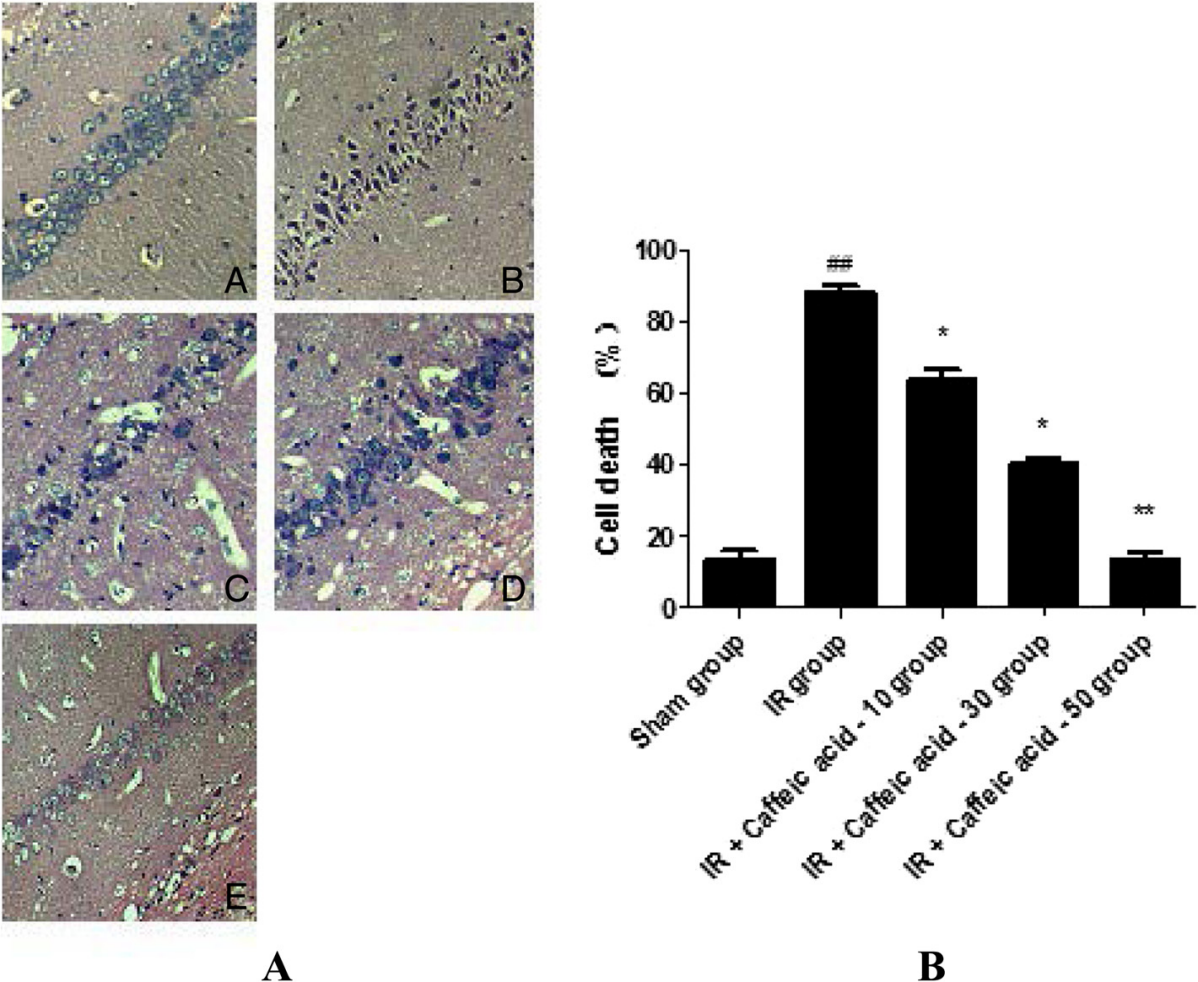
Histopathology of rat hippocampus (HE×400). **A** **:** Sham group; B: I/R; C: I/R + Caffeic acid (10 mg · kg^−1^); D: I/R + Caffeic acid (30 mg · kg^−1^); E: I/R + Caffeic acid (50 mg · kg^−1^). **B:** Effects of Caffeic acid on morphological changes of hippocampus neurons of rats after global cerebral ischemia/reperfusion(mean ± SD, n = 4). ^##^P < 0.01 compared with Sham group, ^*^P < 0.05 and ^**^P < 0.01 compared with IR group.

Results showed that the neurons in the control group were closely arranged, well-structured, had a clear and normal cell morphology, and a pyknosis ratio was calculated at (13.3 ± 2.3)%. In the I/R non-treated group, karyopyknosis was evident, the number of neurons was reduced, and pyknosis ratio was (88.2 ± 1.9)%. In the low-dose caffeic acid group, cell injury was still marked, the pyknosis ratio was (63.6 ± 2.8)%, whereas in the high-dose caffeic acid group, hippocampal neuron karyopyknosis was significantly reduced and the pyknosis ratio was (13.3 ± 3.0)% (Figure 1).

### Changes in 5-LO mRNA expressions

**Figure 2:**
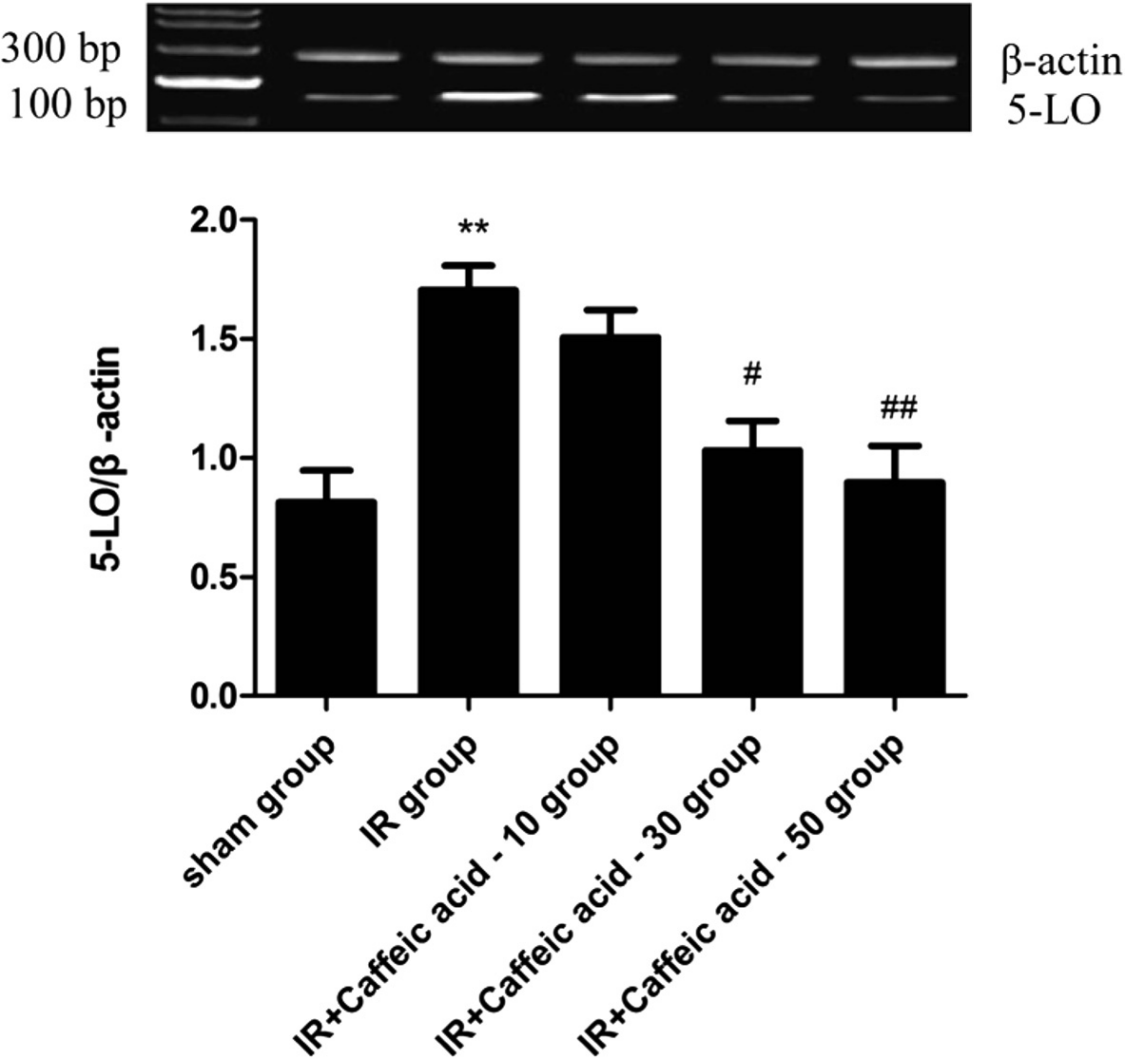
Effects of caffeic acid on 5-LO mRNA expression after global cerebral ischemia and reperfusion injury in rat hippocampus (mean ± SD, n = 9). ^**^ *P* < 0.01 *vs* sham group; ^#^ *P* < 0.05 and ^##^ *P* < 0.01 *vs* IR group.

Results showed that expression of 5-LO mRNA significantly increased in I/R non-treated rat hippocampus (P < 0.01). Caffeic acid significantly reduced the expression of 5-LO mRNA(P < 0.01) dose-dependently in hippocampus (Figure 2).

### Changes in 5-LO protein expressions

**Figure 3:**
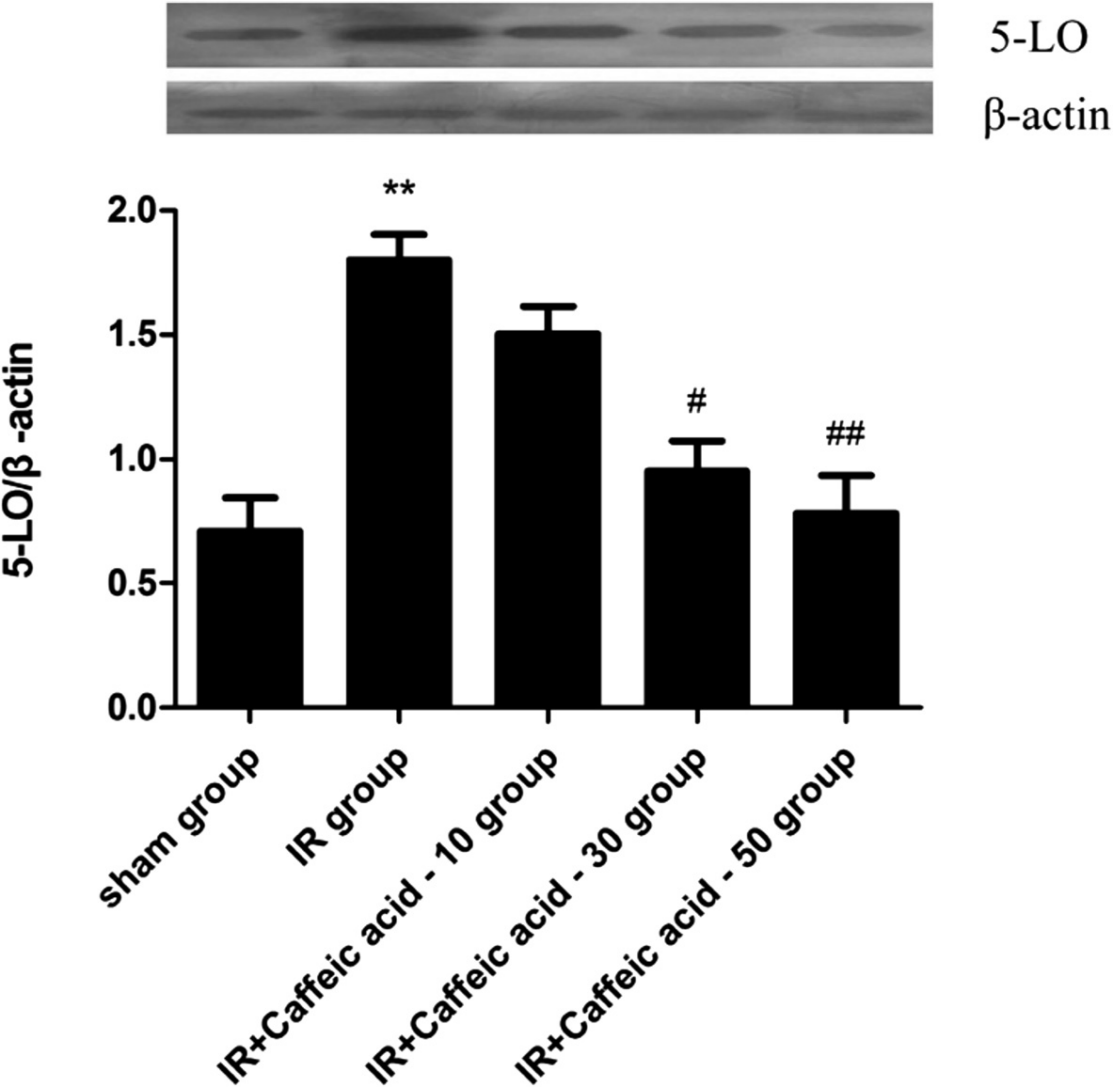
Effects of caffeic acid on 5-LO protein expression after global cerebral ischemia and reperfusion injury in rat hippocampus(mean ± SD, n = 9). ^**^ *P* < 0.01 *vs* sham group; ^#^ *P* < 0.05, ^##^ *P* < 0.01, *vs* model group.

Results showed that global cerebral ischemia-reperfusion injury can lead to a marked increase of 5-LO protein expression (*P* < 0.01) in the hippocampus. Compared with the I/R non-treated group, 5-LO protein expression significantly reduced in the I/R-caffeic acid group (*P* < 0.05 or *P* < 0.01), especially in the I/R-caffeic acid group (50 mg · kg^−1^) (Figure 3).

### Changes in SOD activity and MDA content

Results showed that SOD activity decreased and MDA content increased markedly in the I/R non-treated group when compared with the sham group. The change of MDA levels and SOD activity were antagonized after treatment with caffeic acid, especially with caffeic acid (30 mg · kg^−1^) and caffeic acid (50 mg · kg^−1^) (Table 2).

**Table 2:** **SOD activity and MDA content of rat hippocampus (** $\overline{\mathbf{x}}\pm \mathbf{s}$ ***, n = 4*** **)**

	***SOD***	***MDA***
	***/U · mg*** ^***−1***^ ***Pro***	***/ěmol•g*** ^***−1***^ ***Pro***
Sham	11.05 ± 0.65	0.42 ± 0.04
I/R	7.13 ± 0.38^**^	1.08 ± 0.07^**^
I/R + Caffeic acid (10 mg · kg^−1^)	8.21 ± 0.28^#^	0.91 ± 0.14^#^
I/R + Caffeic acid (30 mg · kg^−1^)	10.30 ± 0.01^##^	0.70 ± 0.05^##^
I/R + Caffeic acid (50 mg · kg^−1^)	10.69 ± 0.64^##^	0.52 ± 0.07^##^

^**^ *P* < 0.01*vs* sham operation group;^#^ *P* < 0.05,^##^ *P* < 0.01*vs* nodel group.

### Changes in protein expression of NF-κBp65

**Figure 4:**
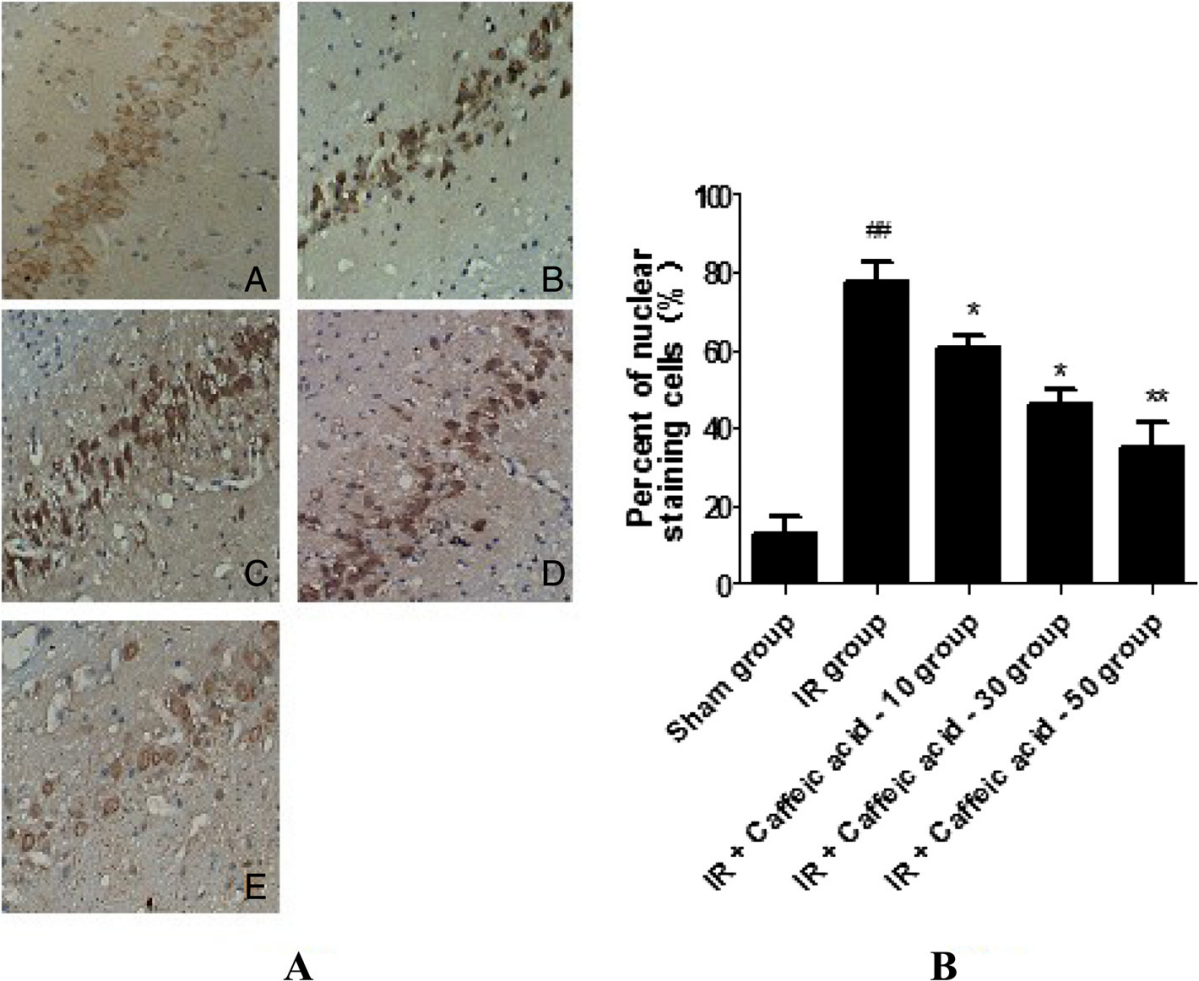
Expression of NF-κBp65in hippocampal neurons of rats (HE × 400). **A** **:** Sham group; B: I/R group; C: I/R + Caffeic acid (10 mg · kg^−1^) group; D: I/R + Caffeic acid(30 mg · kg^−1^) group; E: I/R + Caffeic acid (50 mg · kg^−1^) group. **B:** Effects of Caffeic acid on NF-κBp65 expression in hippocampus of rats after global cerebral ischemia/reperfusion (mean ± SD*,* n = 3).

Immunohistochemistry results showed that the protein expression of NF-κBp65 in the I/R non-treated group was distinctly elevated (77.3 ± 5.5)%, compared with the sham group (12.5 ± 4.6)% (*P* < 0.01). Besides, the protein expression of NF-κBp65 translocated from the cytosol to the nucleus and nucleus were stained to brown in the I/R non-treated group. However, administration of caffeic acid (30 and 50 mg/kg) significantly reduced the global cerebral I/R-induced elevation of NF-κBp65 protein expressions level and increased the cytosolic NF-κBp65 level even though administration of caffeic acid 10 mg/kg had no effect on the global cerebral I/R-induced elevation of NF-κBp65 protein expressions level and the cytosolic NF-κBp65 level (Figure 4).

## Discussion

Caffeic acid is an active component in the phenolic propolis extract and also in a wide variety of plants. It has been reported to have anti-inflammatory, anti-apoptotic, and anti-oxidative properties. In this study, we found that caffeic acid had neuroprotective effects on global cerebral ischemia-reperfusion injury in rats. The study also provides evidence that the mechanism of this protective effect may relate to the inhibition of 5-LO.

Our experimental results showed that global cerebral ischemia-reperfusion impaired learning and memory functions of rats. Compared with the sham group, the time taken to find platform was significantly longer in the I/R non-treated group. Global cerebral ischemia-reperfusion also causes neuronal death and loss of neurons in CA1 subfield of hippocampus. In the sham group, the neurons were closely arranged, well-structured, had a clear and normal cell morphology, and the pyknosis ratio was low. In the I/R non-treated group, the neurons were disorder, the morphology of the neurons was not clear, the number of neurons was reduced, karyopyknosis was evident, and pyknosis ratio was increased significantly. It also elevated 5-LO mRNA, 5-LO protein and NF-κBp65 protein expression. Results suggested a close correlation between global cerebral ischemia-reperfusion injury in rats and 5-LO expression in hippocampus. Similar results were reported [13-16] that ischemia-reperfusion injury increased 5-LO mRNA expression, production of LTC4, translocation of cytosolic 5-LO to membranes, and generation of oxygen radicals. Y. Zhou et al. [56] also found that the 5-LO expression and the enzymatic activity increased after focal cerebral ischemia. Taken together, these results and our findings suggest that there may be a close relationship between overexpression of 5-LO and neuronal damage caused by global cerebral ischemia-reperfusion.

Our results also revealed that caffeic acid, a selective 5-LO inhibitor, protected rats from neuronal damage induced by global cerebral ischemia-reperfusion. It can significantly improve learning and memory function. Caffeic acid antagonized the global cerebral ischemia-reperfusion induced increase in brain MDA levels and the decrease in SOD activity. It downregulated the overexpression of NF-κBp65 protein, as well as 5-LO mRNA and protein expression. The results provided direct evidence that caffeic acid has a significant neuroprotective effect on global cerebral ischemia-reperfusion injury with biochemical and histopathological parameters. These results also suggested that the protective effects of caffeic acid might be related to reduced 5-LO expression [12,28]. The results is consistent with previous reports [57] that minocycline accelerates functional recovery in the chronic phase of focal cerebral ischemia, which may be partly associated with the reduction of 5-LO expression. Similarly, as a 5-LO inhibitor, nordihydroguaiaretic acid also have neuroprotective effect on focal cerebral ischemia-reperfusion injury through inhibiting 5-LO and inflammatory responses mediated by 5-LO metabolites [16].

In this study, we could not explain the mechanism by which caffeic acid reduces 5-LO expression. However, as we know that inflammation and oxidative stress can cause neuronal injury, which further cause the increase of expression of inflammation-related enzyme. On the one hand, the possibility is that caffeic acid reduces reactive oxygen species (ROS) production, which may inhibit 5-LO expression. Our experimental results showed that global cerebral ischemia-reperfusion reduced the activity of SOD (an eliminator of free radicals), increased the content of MDA(an indicator of lipid peroxidation) and NF-κBp65 (an important transcription factor that plays a pivotal role in mediating inflammatory response to pro-inflammatory cytokines and ROS) protein expression. The consequent oxidative stress and overproduction of ROS during ischemia-reperfusion event has a critical role for the level of brain injury [58,59]. There is a lot of evidence in many studies that accumulation of ROS through I/R causes neuronal damage and increases the occurrence of apoptotic cell death in the brain [60-62]. The results also revealed that caffeic acid, a selective 5-LO inhibitor, antagonized the global cerebral ischemia-reperfusion induced increase in brain MDA levels and the decrease in SOD activity. It downregulated the overexpression of NF-κBp65 protein. In a word, caffeic acid ameliorates the oxidative stress following global cerebral ischemia-reperfusion injury. The present study accords with previous reports [63,64]. Caffeic acid reduces reactive oxygen species (ROS) production, which may inhibit 5-LO expression. This hypothesis is supported by the finding that minocycline reduces ROS production, such as peroxyl radicals [65], hydroxyl radicals [66] and superoxide anion, which in turn inhibits 5-LO expression and decreases leukotriene synthesis [67]. On the other hand, caffeic acid could inhibit oxidative stress and reduce the release of inflammatory factor leukotriene synthesis by inhibition of 5-LO. This could decrease the neuronal injury, which in turn caused decrease of the 5-LO expression by feedback regulation.

The mechanisms of the neuroprotective effects of caffeic acid on global cerebral ischemia-reperfusion injury are not fully understood, but the effects can be explained at least in two ways. One is its anti-oxidant and anti-inflammatory ability as shown in lots of studies. Like CAPE, caffeic acid as an antioxidant can scavenge a number of reactive species. In the present study, we confirmed the anti-oxidant ability of caffeic acid after global cerebral ischemia-reperfusion injury. We found that global cerebral ischemia-reperfusion injury reduces the activity of SOD and increases the amount of MDA. These changes can be inhibited by caffeic acid, similar as by edaravone, an anti-oxidant agent with neuroprotective effects [63,64]. Another explanation for the protective effects of caffeic acid is the inhibition of 5-lipoxygenase (5-LO) activity [27]. 5-LO activation is related to cell injury and can be inhibited by caffeic acid. It was reported that 5-LO enzymatic activity was increased and its expression was up-regulated in focal cerebral ischemia, and the increased 5-LO enzymatic activity was inhibited by caffeic acid [12]. Therefore, caffeic acid might attenuate astrocyte proliferation and glial scar formation via inhibiting 5-LO activity.

Nevertheless, further evidence should be obtained from experimental and clinical studies to confirm our hypothesis. Considering that COX-2 is a similar inflammatory mediator, catalyzing the conversion of arachidonic acid into prostaglandins, it should be of interest to investigate whether a combination of 5-LO and COX-2 inhibitors would exert a more pronounced effect on global cerebral ischemia-reperfusion injury than either inhibitor alone.

## Conclusion

In summary, caffeic acid has neuroprotective effects on global cerebral ischemia-reperfusion injury in rats, as shown by the decrease in MDA overproduction, increase in SOD activity of the hippocampus. The neuroprotective effect is likely to be mediated through the inhibition of 5-LO. These findings suggest that caffeic acid may represent a new prototype compound of potential neuroprotective agents in the treatment of global cerebral ischemia-reperfusion injuries.
